# Comparative study on structural and functional brain differences in mild cognitive impairment patients with tinnitus

**DOI:** 10.3389/fnagi.2024.1470919

**Published:** 2024-09-02

**Authors:** Sang-Yoon Han, Heejung Kim, Yejin Yun, Min Jae Lee, Jun-Young Lee, Sun-Won Park, Yu Kyeong Kim, Young Ho Kim

**Affiliations:** ^1^Department of Otolaryngology-Head and Neck Surgery, College of Medicine, Hanyang University, Seoul, Republic of Korea; ^2^Department of Nuclear Medicine, Seoul National University Boramae Medical Center, Seoul National University College of Medicine, Seoul, Republic of Korea; ^3^Institute of Radiation Medicine, Medical Research Center, Seoul National University, Seoul, Republic of Korea; ^4^Department of Otorhinolaryngology, Seoul National University College of Medicine, Seoul, Republic of Korea; ^5^Department of Biochemistry and Molecular Biology, Seoul National University College of Medicine, Seoul, Republic of Korea; ^6^Department of Psychiatry, Seoul National University College of Medicine and Boramae Medical Center, Seoul, Republic of Korea; ^7^Department of Radiology, Seoul National University College of Medicine, Seoul Metropolitan Government Seoul National University Boramae Medical Center, Seoul, Republic of Korea; ^8^Department of Otorhinolaryngology-Head and Neck Surgery, Boramae Medical Center, SMG-SNU, Seoul, Republic of Korea

**Keywords:** tinnitus, mild cognitive impairment, auditory cortex, amyloid plaque, degenerative change, brain volume, brain metabolism, salience network

## Abstract

**Objective:**

Tinnitus may be associated with various brain changes. However, the degenerative changes in patients with tinnitus have not been extensively investigated. We aimed to evaluate degenerative, structural, and functional brain changes in patients with mild cognitive impairment (MCI) who also suffer from tinnitus.

**Materials and methods:**

This study included participants aged 60 to 80 years with MCI and a hearing level better than 40 dB. The participants were classified into two groups: MCI with tinnitus (MCI-T) and MCI without tinnitus (MCI-NT). All patients underwent Tinnitus Handicap Inventory (THI), 3 T brain MRI, F18-florapronol PET, and F18-FDG PET.

**Results:**

The MCI-T group exhibited higher β-amyloid deposition in the superior temporal gyrus, temporal pole, and middle temporal gyrus compared to the MCI-NT group (*p* < 0.05 for all). Additionally, the MCI-T group showed increased metabolism in the inferior frontal gyrus, insula, and anterior cingulate cortex (ACC) (*p* < 0.005 for all). The THI score was strongly correlated with increased volume in the insula, ACC, superior frontal gyrus, supplementary motor area, white matter near the hippocampus, and precentral gyrus (*p* < 0.05 for all). Moreover, the MCI-T group demonstrated higher metabolic activity in the default mode network (DMN) and lower activity in the executive control network (ECN) (*p* < 0.05 for all). In the MCI-T group, the posterior DMN was positively correlated with the visual network and negatively with the ECN, whereas in the MCI-NT group, it correlated positively with the ECN.

**Conclusion:**

The MCI-T group exhibited greater β-amyloid accumulation in the auditory cortex and more extensive changes across various brain networks compared with the MCI-NT group, potentially leading to diverse clinical symptoms such as dementia with semantic deficits or depression. Tinnitus in MCI patients may serve as a biomarker for degenerative changes in the temporal lobe and alterations in brain network dynamics.

## Introduction

Tinnitus, a chronic condition manifesting as ringing or buzzing sounds, persistently disrupts daily life. It affects approximately 20% of the general population aged 12 and older (Park et al., 2014). Its prevalence is notable among children and adolescents, increasing with age (Park et al., 2014). Tinnitus is divided into subjective, which is undetectable by physicians, and objective, which may originate from adjacent structures such as blood vessels or muscle tremors (Crummer and Hassan, 2004; Henry et al., 2014; Han et al., 2009). Unlike objective tinnitus, the causes of subjective tinnitus (Crummer and Hassan, 2004; Han et al., 2009; Henry et al., 2014) are highly varied. Hearing loss is a primary factor influencing tinnitus (Crummer and Hassan, 2004; Han et al., 2009; Henry et al., 2014). Additionally, subjective tinnitus is associated with depressive moods, physical pain, and sleep quality (Ausland et al., 2021; Hackenberg et al., 2023; Alster et al., 1993).

Recent studies have established a significant association between otologic symptoms and degenerative changes in the brain (Zheng et al., 2022; Bisogno et al., 2021; Cheng et al., 2021; Shulman et al., 2007). Hearing loss can accelerate cognitive impairment by promoting the degeneration of brain function (Zheng et al., 2022; Bisogno et al., 2021). Moreover, these changes can be mitigated by the use of hearing aids (Lin et al., 2023). Lin et al. demonstrated that hearing aids could partially offset cognitive deterioration in a long-term, large-scale cohort study (Lin et al., 2023). Furthermore, a link between degenerative brain changes and tinnitus has also been established by several studies (Chu et al., 2020; Cheng et al., 2021). These studies disclosed that dementia, Parkinsonism, and mild cognitive impairment (MCI) are associated with tinnitus (Chu et al., 2020; Cheng et al., 2021). However, the mechanisms underlying this association remain to be fully elucidated.

Prior research aimed to clarify the identification of tinnitus-associated brain networks. Lee et al. and De Ridder et al. discovered that the default mode network (DMN), central executive network (CEN), and salience network (SN) are implicated in tinnitus, and alterations in these networks can trigger tinnitus (Lee et al., 2022; De Ridder et al., 2022). In addition, activation of the noise-canceling pathway can also provoke tinnitus (Lee et al., 2022; De Ridder et al., 2022). Furthermore, several studies on brain changes in tinnitus patients have shown that tinnitus is linked with alterations in brain structures and metabolism (Lee et al., 2022; De Ridder et al., 2022; Mirz et al., 2000). However, the relationship with other brain degenerative changes, such as β-amyloid accumulation, in tinnitus patients remains unexplored. Although some studies have demonstrated an association between β-amyloid accumulation and hearing loss (Pan et al., 2024; Zheng et al., 2022), they did not evaluate the β-amyloid accumulation pattern in tinnitus patients.

Herein, we aimed to identify the structural and functional brain changes in MCI patients with tinnitus, particularly focusing on degenerative changes including β-amyloid accumulation patterns among MCI patient groups with and without tinnitus in individuals with age-normative hearing levels.

## Materials and methods

### Subjects

We included participants aged between 60 and 80 years who had experienced tinnitus for over six months, were diagnosed with MCI, and had a mean hearing level (average hearing level at 0.5 kHz, 1 kHz, 2 kHz, and 4 kHz) of less than 40 dB. We excluded individuals with severe neurocognitive disorders such as schizophrenia or a history of dementia, severe cerebrovascular disease, brain lesions or trauma, previous brain surgery, those who had abused alcohol or substances within the previous year, those with cognitive impairments or severe deafness that hindered the conduct of surveys, hearing tests, or tinnitus examinations, a history of sudden hearing loss or traumatic hearing injuries, users of hearing aids, those whose illiteracy interfered with neuropsychological testing, and those with metal in their bodies, claustrophobia, or other conditions that precluded MRI imaging. Consequently, we prospectively included 30 patients with MCI, of whom 7 were in the MCI-T group and 23 in the MCI-NT group.

### Ethics

This study was conducted following approval from the Institutional Review Board (IRB No. 20-2019-67), and all participants provided informed consent prior to participation.

### Surveys for evaluation of tinnitus and MCI

The Tinnitus Handicap Inventory (THI) survey (Newman et al., 1996) was administered to assess tinnitus-associated distress and evaluate the otological symptoms of all enrolled subjects. The Korean Version of the Consortium to Establish a Registry for Alzheimer’s Disease (CERAD-K) (Lee et al., 2002) was utilized to assess the cognitive function of the participants.

### Image acquisition and preprocessing

#### MRI acquisition and preprocessing

A Philips 3 T MRI system (Achieva, Philips Medical Systems, Best, The Netherlands) was employed for MRI imaging. High-resolution T1-weighted spoiled gradient recall (SPGR) MRI sequences (TR = 9.9 ms, TE = 4.6 ms, flip angle = 8°, FOV = 320 × 320 mm^2^, voxel size of 0.7 × 0.7 × 0.7 mm) were used. Preprocessing involved the use of Statistical Parametric Mapping software (SPM12, Wellcome Department of Imaging Neuroscience, London, United Kingdom) running in Matlab 9.14. Voxel-based morphometry analysis (VBM) was executed through the CAT12 toolbox in SPM12.^1^ Images were segmented into gray matter (GM), white matter, and cerebrospinal fluid, and nonlinearly normalized to a standard stereotactic space using the DARTEL algorithm. The spatially normalized images were then smoothed with an 8 mm FWHM Gaussian kernel. An absolute threshold value of 0.1 was applied to eliminate artifacts on the gray matter.

#### PET acquisition and preprocessing

Subjects underwent two positron tomography (PET) scans: an F-18 Florapronol (FPN) PET to measure amyloid deposition, and an F-18 fluorodeoxyglucose (FDG) PET to evaluate metabolic uptake in the brain, using a PET-CT scanner (Gemini TF64, Philips Healthcare, Best, the Netherlands), with the scans conducted four weeks apart.

F-18 FDG scans: after injecting 370 MBq or less, FDG emission scans commenced 40 min post bolus injection and continued for 20 min.

F-18 FPN scans: patients received 370 MBq of F-18 florapronol (Alzavue, FutureChem Pharma), followed by the immediate acquisition of dynamic images for 10 min. Delayed imaging sessions began 30 min post-injection.

Preprocessing involved using SPM12 to linearly coregister PET images to individual T1-MRI scans. The aligned FPN and FDG PET images were then spatially transformed to conform to the MNI (Montreal Neurological Institute) spatial template. The spatially normalized FDG PET image was smoothed with a Gaussian kernel of 12 mm FWHM, and the images were scaled to the global mean to minimize individual variations.

To quantify amyloid deposition on FPN PET, the standardized uptake value ratio (SUVR) was used to normalize signal intensity to the total cerebellar GM; the resulting SUVR images were also smoothed with a Gaussian kernel of 12 mm FWHM.

#### Independent component analysis

For FDG PET, independent component analysis (ICA) was conducted using the GIFT toolbox (version 3.0a; Medical Imaging Analysis Lab, The Mind Research Network; http://mialab.mrn.org/software/gift). The analysis of individually smoothed PET images identified components showing common subject covariation. A total of twenty-four components were extracted from the FDG PET data, with spatial correlation maps being generated using a *z*-score threshold of 2.0. Components considered meaningful based on visual screening presented major clusters primarily in the gray matter (Yakushev et al., 2013). A measure termed “loading coefficients” was used to gauge the degree of component expression in individual subjects.

### Statistical analysis

Statistical analysis entailed the use of general linear modeling via SPM12. Voxel-wise statistics for VBM, FDG PET, and FPN PET were derived using non-parametric permutation-based testing (5,000 permutations) and threshold-free cluster enhancement (TFCE). The significance level was set at FWE *p* < 0.05. Analysis for VBM was controlled for age, sex, and total intracranial volume (TIV), while PET analyses were adjusted for age and sex as covariates of no interest. For the ICA analysis, network integrity indices between groups were compared using a 2-sample *t*-test, setting the alpha value at 0.05.

## Results

### Demographics, CERAD-K, hearing level, and THI-score

The age of the MCI-T group was 71.0 ± 6.6, which did not differ significantly from that of the MCI-NT group (74.1 ± 4.7, *p* = 0.173) (Table 1). Additionally, the gender distribution in the MCI-T group (M:F = 5:2) was comparable to that in the MCI-NT group (M:F = 18:5, *p* = 1.000) (Table 1). In the MCI-T group, only one subject was ambidextrous, whereas all other participants in both the MCI-T and MCI-NT groups were right-handed. Moreover, the MCI-T group attained higher scores on the CERAD-K than the MCI-NT group (MCI-T: 63.4 ± 11.4; MCI-NT: 52.8 ± 11.3, *p* = 0.04). The hearing level of MCI-T group (22.1 ± 8.0 dB) was not different compared to MCI-NT group (26.4 ± 6.3 dB, *p* = 0.145). Additionally, the THI score in the MCI-T group was 34.3 ± 32.4 (Table 1). In contrast, the THI score for the MCI-NT group was reported as 0.

**Table 1:** Subject demographics.

	Age (Mean ± SD)	Gender (M:F)	CERAD-K (score)	Hearing level (dB)	THI (score)
MCI-T (*N* = 7)	71.0 ± 6.6	5:2	63.4 ± 11.4	22.1 ± 8.0	34.3 ± 32.4
MCI-NT (*N* = 23)	74.1 ± 4.7	18:5	52.8 ± 11.3	26.4 ± 6.3	0
*p*-value	0.173	1.000	0.04	0.145	–

SD, standard deviation; M, male; F, female; CERAD-K, Korean version of the Consortium to Establish a Registry for Alzheimer’s Disease Assessment Packet; THI, tinnitus handicap inventory; MCI, mild cognitive impairment; T, tinnitus; NT, non-tinnitus.

### β-amyloid deposition of brain in MCI-T and MCI-NT group

**Figure 1:**
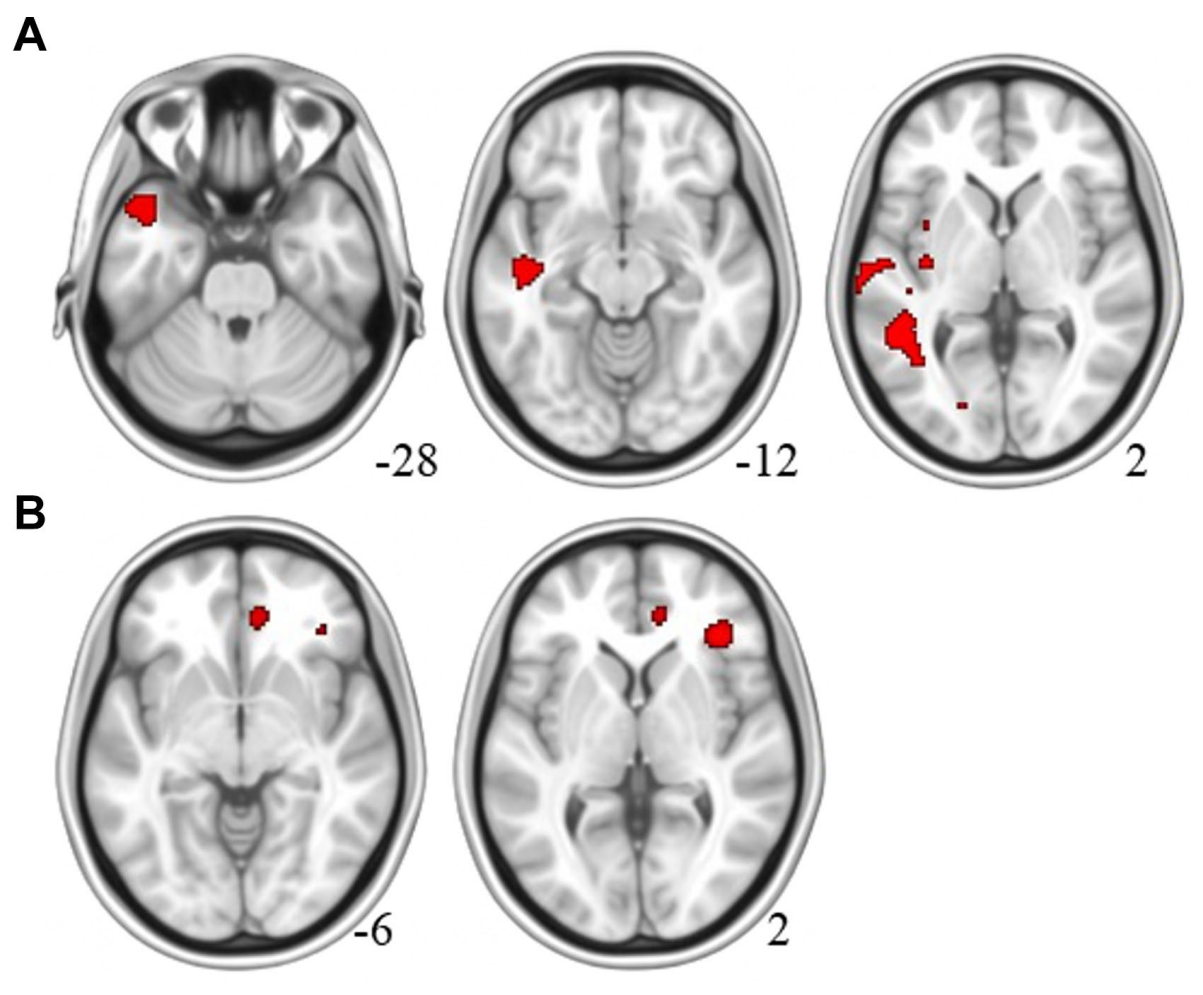
Brain region of the MCI-T group with **(A)** higher β-amyloid plaque accumulation in F-18 florapronol PET and **(B)** greater glucose metabolism than the MCI-NT Group in F-18 FDG-PET.

Compared to the MCI-NT group, the MCI-T group showed significantly higher uptake in F-18 FPN PET [TFCE, FWE *p* < 0.05, cluster size (*k*) > 100] in the left superior and middle temporal gyrus, as well as the temporal pole (Figure 1A; Supplementary Figure S1A; Table 2). Meanwhile, the MCI-NT group showed no regions of significantly higher uptake compared to the MCI-T group.

**Table 2:** Brain regions showing significant group differences.

		L/R	Clusters		MNI Coordinates		
	Regions		(voxels)	TFCE	*x*	*y*	*z*
A. Amyloid deposition group differences							
MCI-T > MCI-NT	Middle temporal gyrus	L	2,195	1,192	−48	−46	2
	Temporal pole/superior temporal gyrus	L	190	1,144	−48	16	−28
	Superior temporal gyrus	L	167	1,141	−46	−16	−12
B. Glucose metabolism group differences							
MCI-T > MCI-NT	Inferior frontal cortex/insula	R	137	1,417	38	32	2
	Anterior cingulate cortex (ACC)	R	339	1,248	8	40	−6

MCI, mild cognitive impairment; T, tinnitus; NT, non-tinnitus; the statistical threshold was non-parametric statistics, based on threshold-free cluster enhancement (TFCE), FWE *p* < 0.05, with a cluster threshold of 100 voxels.

### Gray matter volume and metabolic activity changes of brain in MCI-T and MCI-NT group

For group comparisons using VBM, no significant difference was observed in gray matter volume between the MCI-T and MCI-NT groups [TFCE, FWE *p* < 0.05, cluster size (*k*) > 100]. However, glucose metabolism was significantly higher in the MCI-T group [TFCE, FWE *p* < 0.05, cluster size (*k*) > 100] in the right inferior frontal cortex and ACC (Figure 1B; Supplementary Figure S1B; Table 2). No region showed greater glucose metabolism in the MCI-NT group compared to the MCI-T group.

**Figure 2:**
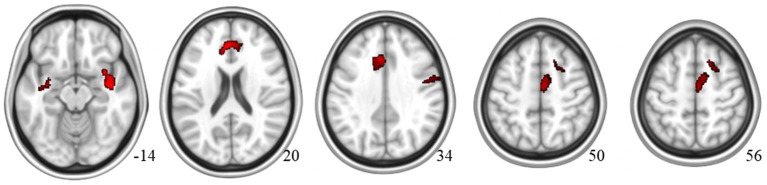
Brain regions showing significant GM volume changes associated with THI score in MRI.

For the correlation analysis with THI score, it was found to be positively associated with gray matter volume in the insula, ACC/superior frontal gyrus, supplementary motor area, and precentral area [TFCE, uncorrected *p* < 0.005, cluster size (*k*) > 100] (Figure 2; Supplementary Figure S2; Table 3).

**Table 3:** Brain regions demonstrating significant GM volume correlations with THI score differences between MCI-T and MCI-NT groups.

		L/R	Clusters		MNI Coordinates		
	Regions		(voxels)	TFCE	*x*	*y*	*z*
Gray matter volumes correlated with THI							
Positive	Superior temporal gyrus/insula	R	797	1,220	42	−9	−14
	ACC/superior frontal gyrus	L	957	1,162	−3	39	20
	Supplementary motor area (SMA)	R	459	855	4	−8	50
	White matter (near the hippocampus)	L	196	818	−34	−8	−14
	Superior frontal gyrus	R	140	679	20	20	56
	Precentral gyrus	R	337	544	51	−3	34

THI, tinnitus handicap inventory; ACC, anterior cingulate cortex; the statistical threshold was non-parametric statistics, based on threshold-free cluster enhancement (TFCE), uncorrected *p* < 0.005, with a cluster threshold of 100 voxels.

### Independent component analysis

**Figure 3:**
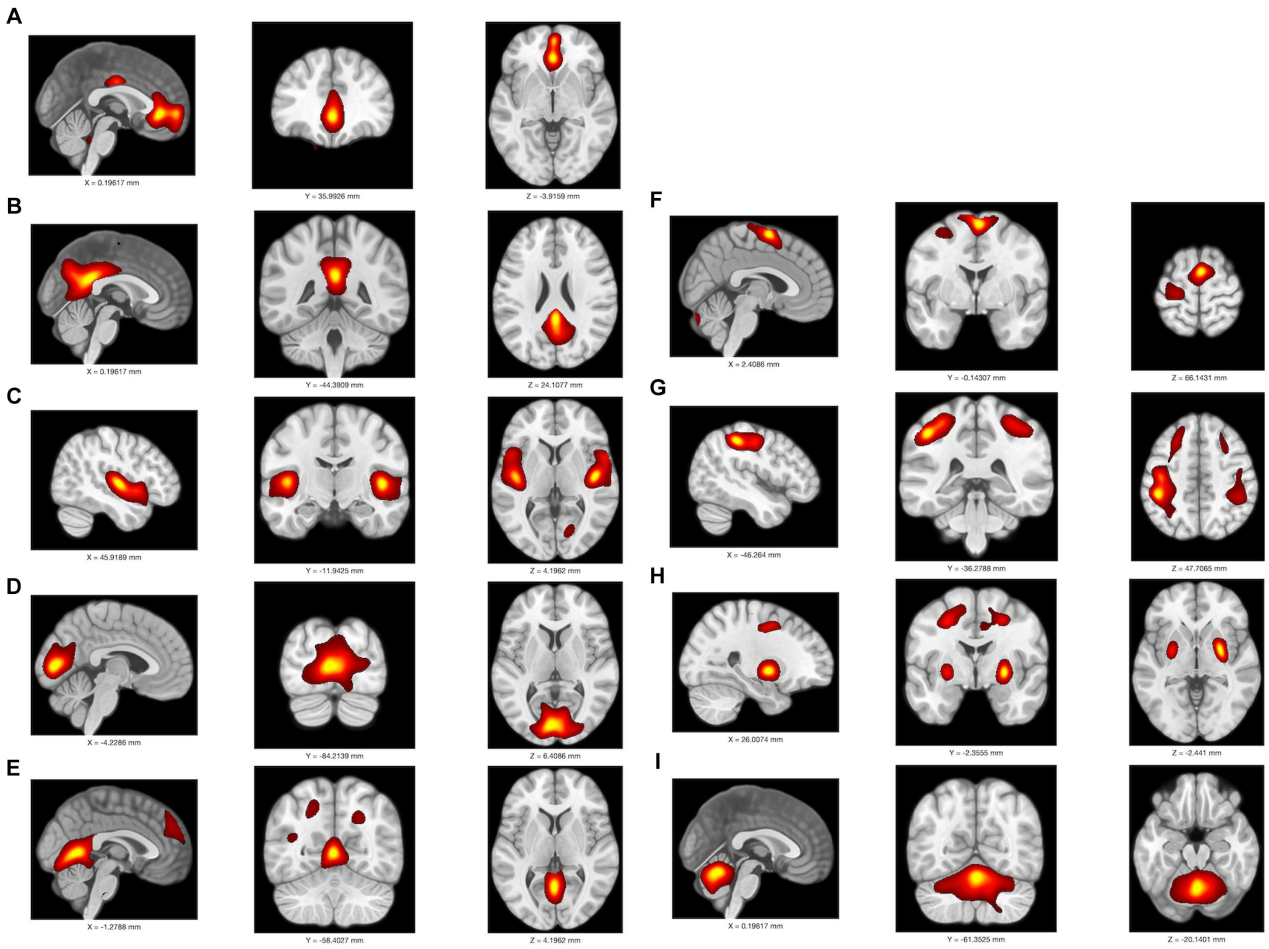
An overlay is displayed of the independent component map at a threshold of *z* > 2.0 on a T1 template in MNI space in F-18 FDG-PET; it includes **(A)** anterior DMN, **(B)** posterior DMN, **(C)** auditory network; AUN, **(D–E)** primary visual network; pVIS, **(F)** somatosensory network; SMN, **(G)** executive control network; ECN, **(H)** basal ganglia network; BGN, **(I)** cerebellar network; CBN.

**Figure 4:**
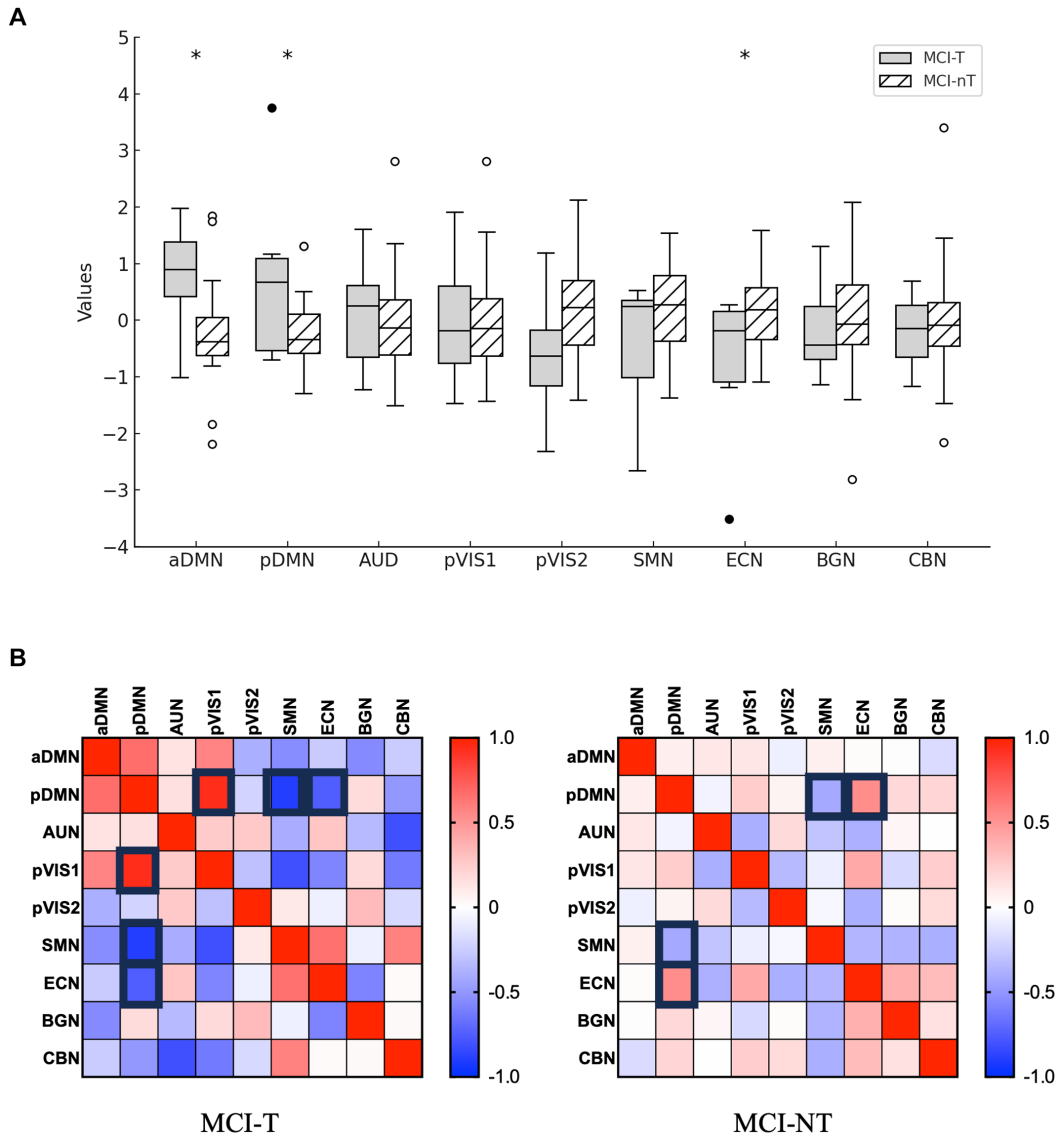
Distribution of network integrity indices is shown. **(A)** The boxplots depict loading coefficients for F-18 FDG-PET. *p < 0.05. **(B)** The correlation matrix (with strong rectangle lines) represents pairs with a threshold *p* < 0.05.

Among 24 independent components, 9 were identified as meaningful components in FDG PET data. Spatial maps included the anterior and posterior default mode network (DMN), auditory network (AUN), visual network (VIN), somatosensory network (SMN), cerebellar network (CBN), basal ganglia network (BGN), and executive control network (ECN) as depicted in Figure 3. The ICA results indicated notably lower integrity of the ECN and higher integrity of both the anterior and posterior DMN in the MCI-T group compared to the MCI-NT group (Figure 4A). In the connectivity matrix, there was a significantly positive correlation between the VIN and the posterior DMN in the MCI-T group, a correlation absent in the MCI-NT group (Figure 4B). Additionally, the ECN and pDMN displayed a negative correlation in the MCI-T group, whereas a positive correlation was noted in the MCI-NT group.

## Discussion

We assessed β-amyloid plaque accumulation, brain volume, and metabolism in relation to the presence of tinnitus in MCI patients. Our findings indicated distinct differences in brain pathology, structure, and function between the MCI-T and MCI-NT groups. The MCI-T group exhibited higher β-amyloid plaque deposition particularly in the auditory cortex regions and the temporal lobe. Furthermore, this group demonstrated elevated metabolism in the inferior frontal gyrus, insula, and ACC. Similarly, metabolic activity was higher in the anterior and posterior DMN but reduced in the ECN when compared to the MCI-NT group. The THI scores correlated significantly with the gray matter volume in the insula, ACC, superior frontal gyrus, supplementary motor area, white matter adjacent to the hippocampus, and precentral gyrus. These results highlight structural and functional brain alterations in MCI-T patients, including amyloid plaque deposition in the auditory cortex, increased volume and activity in the DMN, enhanced volume in the SN including the insula, ACC, and parts of the inferior frontal gyrus, and diminished activity in the ECN compared to the MCI-NT group.

β-amyloid plaque contributes to degenerative changes in the brain including neuronal loss (DeTure and Dickson, 2019), with our research demonstrating its deposition in the superior and middle temporal gyrus as well as in the temporal pole regions in the MCI-T group. The affected superior temporal gyrus areas are Brodmann areas 41 and 42, which include the primary auditory cortex, while parts of the Rolandic operculum also belong to the auditory cortex (Khalighinejad et al., 2021; Moerel et al., 2014). Since tinnitus is linked to aberrant auditory cortex activity (Galazyuk et al., 2012; Lee et al., 2022) and β-amyloid plaque deposition can induce abnormal neuronal activity in adjacent cells (Zott et al., 2019; Wood, 2019), the presence of β-amyloid plaque in parts of the auditory cortex may contribute to the onset of tinnitus in these cortical areas.

Additionally, the middle temporal gyrus is associated with semantic memory, visual perception, and multi-sensory integration (Onitsuka et al., 2004; Mesulam, 1998), which previous studies have demonstrated to be diminished in patients with tinnitus (Karimi Boroujeni et al., 2020; Mahoney et al., 2011; Dehmel et al., 2008; Lanting et al., 2010; Spiegel et al., 2015). Moreover, the temporal pole is implicated in a range of functions including language, semantic and auditory processing, visual processing, multimodal sensory integration, and emotion (Herlin et al., 2021). The β-amyloid plaque-deposited areas in the MCI-T group were predominantly in regions associated with semantic processing and visual or auditory sensory functions. Consequently, the pattern and progression of cognitive impairment in the MCI-T group might differ from that in MCI-NT patients, characterized initially by multi-sensory deficits coupled with semantic impairments. Thus, tinnitus in the MCI group might be considered a biomarker indicating a predisposition towards dementia with semantic deficits.

Higher metabolic activity of glucose in the SN, encompassing the ACC, insula, and parts of the inferior frontal gyrus, was evident in the MCI-T group, as demonstrated by our findings (Seeley et al., 2007). Additionally, the volume of these areas exhibited a significant correlation with THI. The SN is linked to rewards, motivation, emotion, and pain, and facilitates the switch between the ECN and (Schimmelpfennig et al., 2023). Abnormally activated SN has been identified as one of the chronic tinnitus-related brain network changes (De Ridder et al., 2011; Lee et al., 2022). Our results support the involvement of SN in tinnitus, consistent with previous studies (De Ridder et al., 2011; Lee et al., 2022).

The volume of the white matter adjacent to the hippocampus and superior frontal gyrus was significantly negatively associated with THI. These regions are crucial for learning and memory, functions that are compromised in Alzheimer’s disease (Boisgueheneuc et al., 2006; Yang et al., 2019; Bird and Burgess, 2008). Moreover, the volume of the precentral gyrus and supplementary motor area correlated with the THI score. Earlier studies have shown that motor functions decline as Alzheimer’s disease progresses (Buchman and Bennett, 2011). Another study indicated that the supplementary motor area is also affected in the early stages of Alzheimer’s disease (Vidoni et al., 2012). Given that the THI scores of the MCI-NT group were considered to be 0, the associations between the volumes of these regions and THI might be attributed to the impairments in these areas within the MCI-NT group, suggesting further advanced cognitive and motor function deterioration.

The regions with heightened metabolic activity in the MCI-T group included the DMN. Comprising the dorsal medial prefrontal cortex, posterior cingulate cortex, precuneus, and angular gyrus, as well as the medial orbitofrontal cortex and anterior cingulate cortex (Yeshurun et al., 2021), the DMN is active during rest, exploration, and unfocused states (Yeshurun et al., 2021). Increased activity of the DMN has been documented in prior studies (Chen et al., 2018a; Chen et al., 2018b). The heightened activity of the DMN accounts for the predominance of tinnitus perception during rest (Chen et al., 2018a; Chen et al., 2018b). Our results align with these earlier findings.

Additionally, our metabolic network analysis showed that the MCI-T group exhibited higher DMN activity and lower ECN activity compared to the MCI-NT group. The ECN, also associated with tinnitus (De Ridder et al., 2022) governs executive functions critical for goal-oriented actions, such as working memory and attention (De Ridder et al., 2022; Vincent et al., 2008). This network comprises the lateral prefrontal cortex, anterior cingulate cortex, and inferior parietal lobule (Vincent et al., 2008). Typically, disruptions involving increased metabolic activity and functional connectivity in these networks have been identified as changes linked to tinnitus in the general population, findings that differ from those of our study (De Ridder et al., 2022; Lee et al., 2022). These discrepancies may result from impairments in and alterations of the connections among the SN, DMN, and ECN in MCI (Xue et al., 2021; Chand et al., 2017). Earlier research indicated that the SN is unable to modulate the DMN or ECN effectively in MCI patients (Xue et al., 2021; Chand et al., 2017). Moreover, the DMN shows elevated activity in conditions like semantic memory decline and tinnitus, (Chen et al., 2018a; Chen et al., 2018b; Gardini et al., 2015) while ECN activity may diminish in MCI patients with impaired executive functions (Liu et al., 2021). Furthermore, the DMN is typically anti-correlated with the ECN (Fox et al., 2005). Thus, the heightened DMN function in the MCI-T group could suppress the ECN, resulting from inadequate modulation by the SN and the strong anti-correlation with the highly active DMN.

In addition, the ICA results indicated that the DMN was negatively correlated with the ECN in the MCI-T group, whereas it showed a positive correlation in the MCI-NT group. These inter-network modifications could stem from alterations in brain networks related to MCI. A preceding study found that increased functional connectivity between the posterior DMN and ECN correlates with greater β-amyloid deposition and reduced working memory (Zhukovsky et al., 2023). In this study, the MCI-NT group, which had a lower CERAD-K score indicating further progression of MCI, exhibited increased connectivity between the posterior DMN and ECN.

The ICA results highlighted that the posterior DMN and posterior visual network were positively correlated in the MCI-T group. Previous research suggested that a positive relationship between the DMN and the visual network reflects prolonged emotional experiences (Xu et al., 2023). Given that some tinnitus patients suffer from emotion-related disorders, such as depression or anxiety (Hackenberg et al., 2023), this positive correlation between the DMN and visual networks might also elucidate the emotional alterations linked to tinnitus.

In addition, the negative correlation between the DMN and SMN appeared to be weaker in the MCI-T group compared to the MCI-NT group. The DMN is typically anti-correlated with the SMN in healthy individuals (Schwarz et al., 2013). The DMN and SMN are important for perception and internal mental activities, respectively (Xie et al., 2024). A previous study reported that a weakened network between the DMN and SMN is present in tinnitus patients, indicating decreased cognitive control and reduced attention (Xie et al., 2024). This study also supports those findings. Consequently, MCI patients with tinnitus may have less attention and cognitive control compared to those without tinnitus.

This study has some limitations. First, the small number of subjects in the experiment group (MCI-T) was a significant constraint. We were unable to enroll enough participants during the COVID-19 era. Additionally, to minimize the effect of covariates such as other coexisting neurocognitive diseases and hearing-related factors, which could influence the brain function, structures, and tinnitus (Noble, 2008; Shinden et al., 2021; Zheng et al., 2022), only tinnitus patients without other neurocognitive diseases and with hearing levels less than 40 dB were included. This resulted in more homogeneous subjects, although the small number of patients was included. To overcome this limitation, we applied nonparametric analysis and used a strict cut-off for significant differences in the various imaging tests. Despite this limitation, our findings consistently demonstrated changes related to tinnitus and THI, which might provide valuable insights into structural and metabolic alterations in the brain cortex and networks.

Additionally, the CERAD-K scores of each group were not equally distributed. Although Alzheimer’s disease was excluded, the degree of MCI might differ between the groups. To adjust for the differences in CERAD-K scores between the groups, we employed multivariable analysis, controlling for CERAD-K as a covariate. This adjustment is an important consideration for future research.

Another limitation of our study was the evaluation of the THI score with a small number of MCI-T patients. The THI score-associated changes of brain volume may reflect the differences between the MCI-T group and the MCI-NT group, rather than gradual changes in each brain region according to the THI score, due to the limited number of subjects. Further studies with a larger MCI-T group may provide a clearer understanding of the THI-associated brain changes.

Since patients with hearing levels greater than 40 dB were not included, this study may not represent the functional or structural changes in chronic tinnitus patients with hearing loss. Further studies evaluating brain changes in chronic tinnitus patients with hearing loss may be necessary.

## Conclusion

The MCI-T group showed significantly more β-amyloid accumulation in the temporal lobe, including the auditory cortex. Additionally, there were volume and metabolic changes in brain regions of the ECN, DMN, and SN associated with tinnitus. These changes included higher functional connectivity in the DMN, increased brain volume in certain parts of the SN, and lower functional connectivity in the ECN. These findings suggest that MCI-T patients demonstrate distinct degenerative patterns in the brain and altered functional connectivity in networks associated with emotions compared to MCI-NT patients. Tinnitus in the MCI group may serve as a biomarker for degenerative changes in the temporal lobe, potentially leading to dementia with semantic deficits, and for increased functional connectivity in emotion-associated brain networks.

## Data Availability

The raw data supporting the conclusions of this article will be made available by the authors, without undue reservation.

## Footnotes

^1^http://neuro.unijena.de/cat

## References

[ref1] Alster J. Shemesh Z. Ornan M. Attias J. (1993). Sleep disturbance associated with chronic tinnitus. Biol. Psychiatry 34, 84–90. doi: 10.1016/0006-3223(93)90260-k 8373941

[ref2] Ausland J. H. Engdahl B. Oftedal B. Steingrïmsdõttir Ó. A. Nielsen C. S. Hopstock L. A.. (2021). Tinnitus and associations with chronic pain: the population-based Tromsø study (2015–2016). PLoS One 16:e0247880. doi: 10.1371/journal.pone.0247880 33651844 PMC7924755

[ref3] Bird C. M. Burgess N. (2008). The hippocampus and memory: insights from spatial processing. Nat. Rev. Neurosci. 9, 182–194. doi: 10.1038/nrn2335, PMID: 18270514

[ref4] Bisogno A. Scarpa A. Di Girolamo S. De Luca P. Cassandro C. Viola P.. (2021). Hearing loss and cognitive impairment: epidemiology, common pathophysiological findings, and treatment considerations. Life 11:e1102. doi: 10.3390/life11101102 PMC8538578 34685474

[ref5] Boisgueheneuc F. D. Levy R. Volle E. Seassau M. Duffau H. Kinkingnehun S.. (2006). Functions of the left superior frontal gyrus in humans: a lesion study. Brain 129, 3315–3328. doi: 10.1093/brain/awl244, PMID: 16984899

[ref6] Buchman A. S. Bennett D. A. (2011). Loss of motor function in preclinical Alzheimer's disease. Expert. Rev. Neurother. 11, 665–676. doi: 10.1586/ern.11.57, PMID: 21539487 PMC3121966

[ref7] Chand G. B. Wu J. Hajjar I. Qiu D. (2017). Interactions of the salience network and its subsystems with the default-mode and the central-executive networks in normal aging and mild cognitive impairment. Brain Connect. 7, 401–412. doi: 10.1089/brain.2017.0509, PMID: 28707959 PMC5647507

[ref8] Chen Y. C. Chen H. Bo F. Xu J. J. Deng Y.. (2018a). Tinnitus distress is associated with enhanced resting-state functional connectivity within the default mode network. Neuropsychiatr. Dis. Treat. 14, 1919–1927. doi: 10.2147/NDT.S164619, PMID: 30122924 PMC6078076

[ref9] Chen Y. C. Liu S. Lv H. Bo F. Feng Y. Chen H.. (2018b). Abnormal resting-state functional connectivity of the anterior cingulate cortex in unilateral chronic tinnitus patients. Front. Neurosci. 12:9. doi: 10.3389/fnins.2018.00009 29410609 PMC5787069

[ref10] Cheng Y.-F. Xirasagar S. Yang T.-H. Wu C.-S. Kao Y.-W. Lin H.-C. (2021). Risk of early-onset dementia among persons with tinnitus: a retrospective case–control study. Sci. Rep. 11:13399. doi: 10.1038/s41598-021-92802-y 34183724 PMC8238939

[ref11] Chu H.-T. Liang C. S. Yeh T.-C. Hu L.-Y. Yang A. C.. (2020). Tinnitus and risk of Alzheimer’s and Parkinson’s disease: a retrospective nationwide population-based cohort study. Sci. Rep. 10:12134. doi: 10.1038/s41598-020-69243-0 32699252 PMC7376045

[ref12] Crummer R. W. Hassan G. A. (2004). Diagnostic approach to tinnitus. Am. Fam. Physician 69, 120–126, PMID: 14727828

[ref13] De Ridder D. Elgoyhen A. B. Romo R. Langguth B. (2011). Phantom percepts: tinnitus and pain as persisting aversive memory networks. Proc. Natl. Acad. Sci. USA 108, 8075–8080. doi: 10.1073/pnas.1018466108, PMID: 21502503 PMC3100980

[ref14] De Ridder D. Vanneste S. Song J. J. Adhia D. (2022). Tinnitus and the triple network model: a perspective. Clin Exp Otorhinolaryngol 15, 205–212. doi: 10.21053/ceo.2022.00815, PMID: 35835548 PMC9441510

[ref15] Dehmel S. Cui Y. L. Shore S. E. (2008). Cross-modal interactions of auditory and somatic inputs in the brainstem and midbrain and their imbalance in tinnitus and deafness. Am. J. Audiol. 17, S193–S209. doi: 10.1044/1059-0889(2008/07-0045), PMID: 19056923 PMC2760229

[ref16] Deture M. A. Dickson D. W. (2019). The neuropathological diagnosis of Alzheimer’s disease. Mol. Neurodegener. 14:32. doi: 10.1186/s13024-019-0333-5 31375134 PMC6679484

[ref17] Fox M. D. Snyder A. Z. Vincent J. L. Corbetta M. Van Essen D. C. Raichle M. E. (2005). The human brain is intrinsically organized into dynamic, anticorrelated functional networks. Proc. Natl. Acad. Sci. USA 102, 9673–9678. doi: 10.1073/pnas.0504136102, PMID: 15976020 PMC1157105

[ref18] Galazyuk A. V. Wenstrup J. J. Hamid M. A. (2012). Tinnitus and underlying brain mechanisms. Curr. Opin. Otolaryngol. Head Neck Surg. 20, 409–415. doi: 10.1097/MOO.0b013e3283577b81, PMID: 22931904 PMC3886369

[ref19] Gardini S. Venneri A. Sambataro F. Cuetos F. Fasano F. Marchi M.. (2015). Increased functional connectivity in the default mode network in mild cognitive impairment: a maladaptive compensatory mechanism associated with poor semantic memory performance. J. Alzheimers Dis. 45, 457–470. doi: 10.3233/JAD-142547, PMID: 25547636

[ref20] Hackenberg B. Døge J. O’brien K. Bohnert A. Lackner K. J. Beutel M. E.. (2023). Tinnitus and its relation to depression, anxiety, and stress-a population-based cohort study. J. Clin. Med. 12:31169. doi: 10.3390/jcm12031169 PMC9917824 36769823

[ref21] Han B. I. Lee H. W. Kim T. Y. Lim J. S. Shin K. S. (2009). Tinnitus: characteristics, causes, mechanisms, and treatments. J. Clin. Neurol. 5, 11–19. doi: 10.3988/jcn.2009.5.1.11 19513328 PMC2686891

[ref22] Henry J. A. Roberts L. E. Caspary D. M. Theodoroff S. M. Salvi R. J. (2014). Underlying mechanisms of tinnitus: review and clinical implications. J. Am. Acad. Audiol. 25:e25. doi: 10.3766/jaaa.25.1.2, PMID: 24622858 PMC5063499

[ref23] Herlin B. Navarro V. Dupont S. (2021). The temporal pole: from anatomy to function—a literature appraisal. J. Chem. Neuroanat. 113:101925. doi: 10.1016/j.jchemneu.2021 33582250

[ref24] Karimi Boroujeni M. Mahmoudian S. Jarollahi F. (2020). The investigation of semantic memory deficit in chronic tinnitus: a behavioral report. Braz. J. Otorhinolaryngol. 86, 185–190. doi: 10.1016/j.bjorl.2018.11.003, PMID: 30683563 PMC9422494

[ref25] Khalighinejad B. Patel P. Herrero J. L. Bickel S. Mehta A. D. Mesgarani N. (2021). Functional characterization of human Heschl’s gyrus in response to natural speech. NeuroImage 235:118003. doi: 10.1016/j.neuroimage.2021.118003 33789135 PMC8608271

[ref26] Lanting C. P. De Kleine E. Eppinga R. N. Van Dijk P. (2010). Neural correlates of human somatosensory integration in tinnitus. Hear. Res. 267, 78–88. doi: 10.1016/j.heares.2010.04.006 20430086

[ref27] Lee J. H. Lee K. U. Lee D. Y. Kim K. W. Jhoo J. H. Kim J. H.. (2002). Development of the Korean version of the consortium to establish a registry for Alzheimer's disease assessment packet (Cerad-K): clinical and neuropsychological assessment batteries. J. Gerontol. B Psychol. Sci. Soc. Sci. 57, 47–53. doi: 10.1093/geronb/57.1.p47 11773223

[ref28] Lee S. J. Park J. Lee S.-Y. Koo J.-W. Vanneste S. De Ridder D.. (2022). Triple network activation causes tinnitus in patients with sudden sensorineural hearing loss: a model-based volume-entropy analysis. Front. Neurosci. 16:28776. doi: 10.3389/fnins.2022.1028776 PMC9714300 36466160

[ref29] Lin F. R. Pike J. R. Albert M. S. Arnold M. Burgard S. Chisolm T.. (2023). Hearing intervention versus health education control to reduce cognitive decline in older adults with hearing loss in the USA (achieve): a multicentre, randomised controlled trial. Lancet 402, 786–797. doi: 10.1016/S0140-6736(23)01406-X 37478886 PMC10529382

[ref30] Liu W. Liu L. Cheng X. Ge H. Hu G. Chisolm T.. (2021). Functional integrity of executive control network contributed to retained executive abilities in mild cognitive impairment. Front. Aging Neurosci. 13:710172. doi: 10.3389/fnagi.2021.710172 34899264 PMC8664557

[ref31] Mahoney C. J. Rohrer J. D. Goll J. C. Fox N. C. Rossor M. N. Warren J. D. (2011). Structural neuroanatomy of tinnitus and hyperacusis in semantic dementia. J. Neurol. Neurosurg. Psychiatry 82, 1274–1278. doi: 10.1136/jnnp.2010.235473, PMID: 21531705 PMC3188784

[ref32] Mesulam M. M. (1998). From sensation to cognition. Brain 121, 1013–1052. doi: 10.1093/brain/121.6.1013 9648540

[ref33] Mirz F. Gjedde A. Ishizu K. Pedersen C. B. (2000). Cortical networks subserving the perception of tinnitus—a PET study. Acta Otolaryngol. Suppl. 543, 241–243. doi: 10.1080/000164800454503, PMID: 10909031

[ref34] Moerel M. De Martino F. Formisano E. (2014). An anatomical and functional topography of human auditory cortical areas. Front. Neurosci. 8:e225. doi: 10.3389/fnins.2014.00225 PMC4114190 25120426

[ref35] Newman C. W. Jacobson G. P. Spitzer J. B. (1996). Development of the tinnitus handicap inventory. Arch. Otolaryngol. Head Neck Surg. 122, 143–148. doi: 10.1001/archotol.1996.01890140029007, PMID: 8630207

[ref36] Noble W. (2008). Treatments for tinnitus. Trends Amplif. 12, 236–241. doi: 10.1177/1084713808320552, PMID: 18635586 PMC4134891

[ref37] Onitsuka T. Shenton M. E. Salisbury D. F. Dickey C. C. Kasai K. Toner S. K.. (2004). Middle and inferior temporal gyrus gray matter volume abnormalities in chronic schizophrenia: an Mri study. Am. J. Psychiatry 161, 1603–1611. doi: 10.1176/appi.ajp.161.9.1603 15337650 PMC2793337

[ref38] Pan L. Li C. Meng L. Zhang G. Zou L. Tian Y.. (2024). Gdf1 ameliorates cognitive impairment induced by hearing loss. Nature Aging 4, 568–583. doi: 10.1038/s43587-024-00592-5, PMID: 38491289

[ref39] Park K. H. Lee S. H. Koo J. W. Park H. Y. Lee K. Y. Choi Y. S.. (2014). Prevalence and associated factors of tinnitus: data from the Korean National Health and nutrition examination survey 2009-2011. J. Epidemiol. 24, 417–426, PMID: 24953134 10.2188/jea.JE20140024 PMC4150014

[ref40] Schimmelpfennig J. Topczewski J. Zajkowski W. Jankowiak-Siuda K. (2023). The role of the salience network in cognitive and affective deficits. Front. Hum. Neurosci. 17:1133367. doi: 10.3389/fnhum.2023.1133367 37020493 PMC10067884

[ref41] Schwarz A. J. Gass N. Sartorius A. Risterucci C. Spedding M. Schenker E.. (2013). Anti-correlated cortical networks of intrinsic connectivity in the rat brain. Brain Connect. 3, 503–511. doi: 10.1089/brain.2013.0168, PMID: 23919836 PMC3796325

[ref42] Seeley W. W. Menon V. Schatzberg A. F. Keller J. Glover G. H. Kenna H.. (2007). Dissociable intrinsic connectivity networks for salience processing and executive control. J. Neurosci. 27, 2349–2356, PMID: 17329432 10.1523/JNEUROSCI.5587-06.2007 PMC2680293

[ref43] Shinden S. Suzuki N. Oishi N. Suzuki D. Minami S. Ogawa K. (2021). Effective sound therapy using a hearing aid and educational counseling in patients with chronic tinnitus. Auris Nasus Larynx 48, 815–822. doi: 10.1016/j.anl.2021.01.001, PMID: 33461856

[ref44] Shulman A. Goldstein B. Strashun A. M. (2007). Central nervous system neurodegeneration and tinnitus: a clinical experience. Int Tinnitus J 13, 118–131, PMID: 18229791

[ref45] Spiegel D. P. Linford T. Thompson B. Petoe M. A. Kobayashi K. Stinear C. M.. (2015). Multisensory attention training for treatment of tinnitus. Sci. Rep. 5:10802. doi: 10.1038/srep10802 26020589 PMC4447068

[ref46] Vidoni E. D. Thomas G. P. Honea R. A. Loskutova N. Burns J. M. (2012). Evidence of altered corticomotor system connectivity in early-stage Alzheimer's disease. J. Neurol. Phys. Ther. 36, 8–16. doi: 10.1097/NPT.0b013e3182462ea6, PMID: 22333920 PMC3288781

[ref47] Vincent J. L. Kahn I. Snyder A. Z. Raichle M. E. Buckner R. L. (2008). Evidence for a frontoparietal control system revealed by intrinsic functional connectivity. J. Neurophysiol. 100, 3328–3342. doi: 10.1152/jn.90355.2008, PMID: 18799601 PMC2604839

[ref48] Wood H. (2019). Glutamate perpetuates amyloid-β-dependent neuronal hyperactivity. Nat. Rev. Neurol. 15, 558–559. doi: 10.1038/s41582-019-0256-3, PMID: 31462750

[ref49] Xie J. Zhang W. Yu C. Wei W. Bai Y. Shen Y.. (2024). Abnormal static and dynamic brain network connectivity associated with chronic tinnitus. Neuroscience 554, 26–33. doi: 10.1016/j.neuroscience.2024.06.034, PMID: 38964452

[ref50] Xu S. Zhang Z. Li L. Zhou Y. Lin D. Zhang M.. (2023). Functional connectivity profiles of the default mode and visual networks reflect temporal accumulative effects of sustained naturalistic emotional experience. Neuro Image 269:119941. doi: 10.1016/j.neuroimage.2023.119941 36791897

[ref51] Xue C. Sun H. Yue Y. Wang S. Qi W. Hu G.. (2021). Structural and functional disruption of salience network in distinguishing subjective cognitive decline and amnestic mild cognitive impairment. ACS Chem. Neurosci. 12, 1384–1394. doi: 10.1021/acschemneuro.1c00051, PMID: 33825444

[ref52] Yakushev I. Chëtelat G. Fischer F. U. Landeau B. Bastin C. Scheurich A.. (2013). Metabolic and structural connectivity within the default mode network relates to working memory performance in young healthy adults. NeuroImage 79, 184–190. doi: 10.1016/j.neuroimage.2013.04.069 23631988

[ref53] Yang H. Xu H. Li Q. Jin Y. Jiang W. Wang J.. (2019). Study of brain morphology change in Alzheimer's disease and amnestic mild cognitive impairment compared with normal controls. Gen Psychiatr 32:e100005. doi: 10.1136/gpsych-2018-100005 31179429 PMC6551438

[ref54] Yeshurun Y. Nguyen M. Hasson U. (2021). The default mode network: where the idiosyncratic self meets the shared social world. Nat. Rev. Neurosci. 22, 181–192. doi: 10.1038/s41583-020-00420-w 33483717 PMC7959111

[ref55] Zheng M. Yan J. Hao W. Ren Y. Zhou M. Wang Y.. (2022). Worsening hearing was associated with higher β-amyloid and tau burden in age-related hearing loss. Sci. Rep. 12:10493. doi: 10.1038/s41598-022-14466-6 35729211 PMC9212197

[ref56] Zhukovsky P. Coughlan G. Buckley R. Grady C. Voineskos A. N. (2023). Connectivity between default mode and frontoparietal networks mediates the association between global amyloid-β and episodic memory. Hum. Brain Mapp. 44, 1147–1157. doi: 10.1002/hbm.26148, PMID: 36420978 PMC9875925

[ref57] Zott B. Simon M. M. Hong W. Unger F. Chen-Engerer H. J. Frosch M. P.. (2019). A vicious cycle of β amyloid-dependent neuronal hyperactivation. Science 365, 559–565. doi: 10.1126/science.aay0198, PMID: 31395777 PMC6690382

