# Supplementary figures and images for: Comparative study on structural and functional brain differences in mild cognitive impairment patients with tinnitus
Source: Front Aging Neurosci. 2024 Sep 2;16:1470919. doi: 10.3389/fnagi.2024.1470919 (PMC11402673; doi:10.3389/fnagi.2024.1470919)

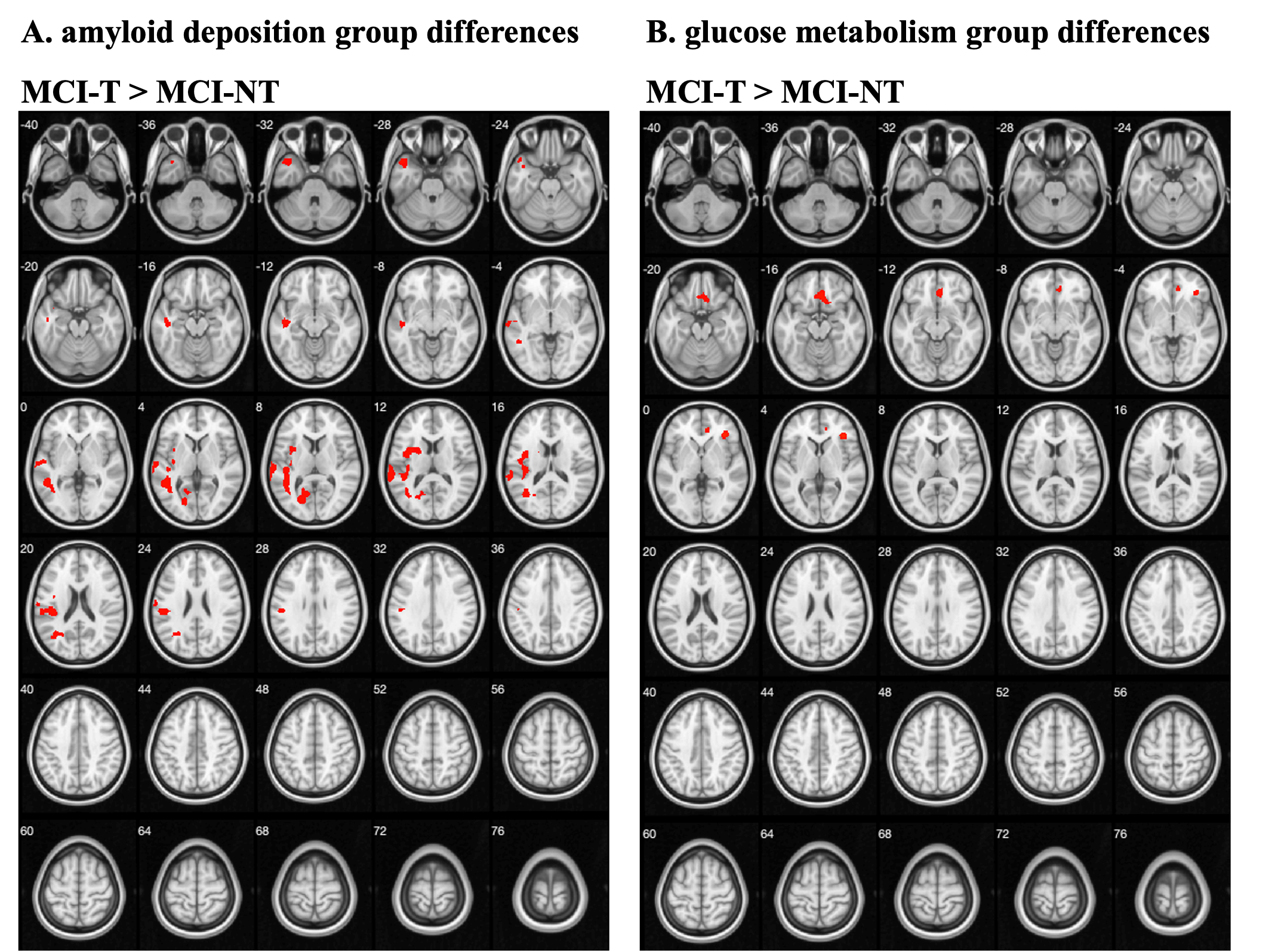

Supplement: Supplementary file 1 [file Image_1.TIFF]

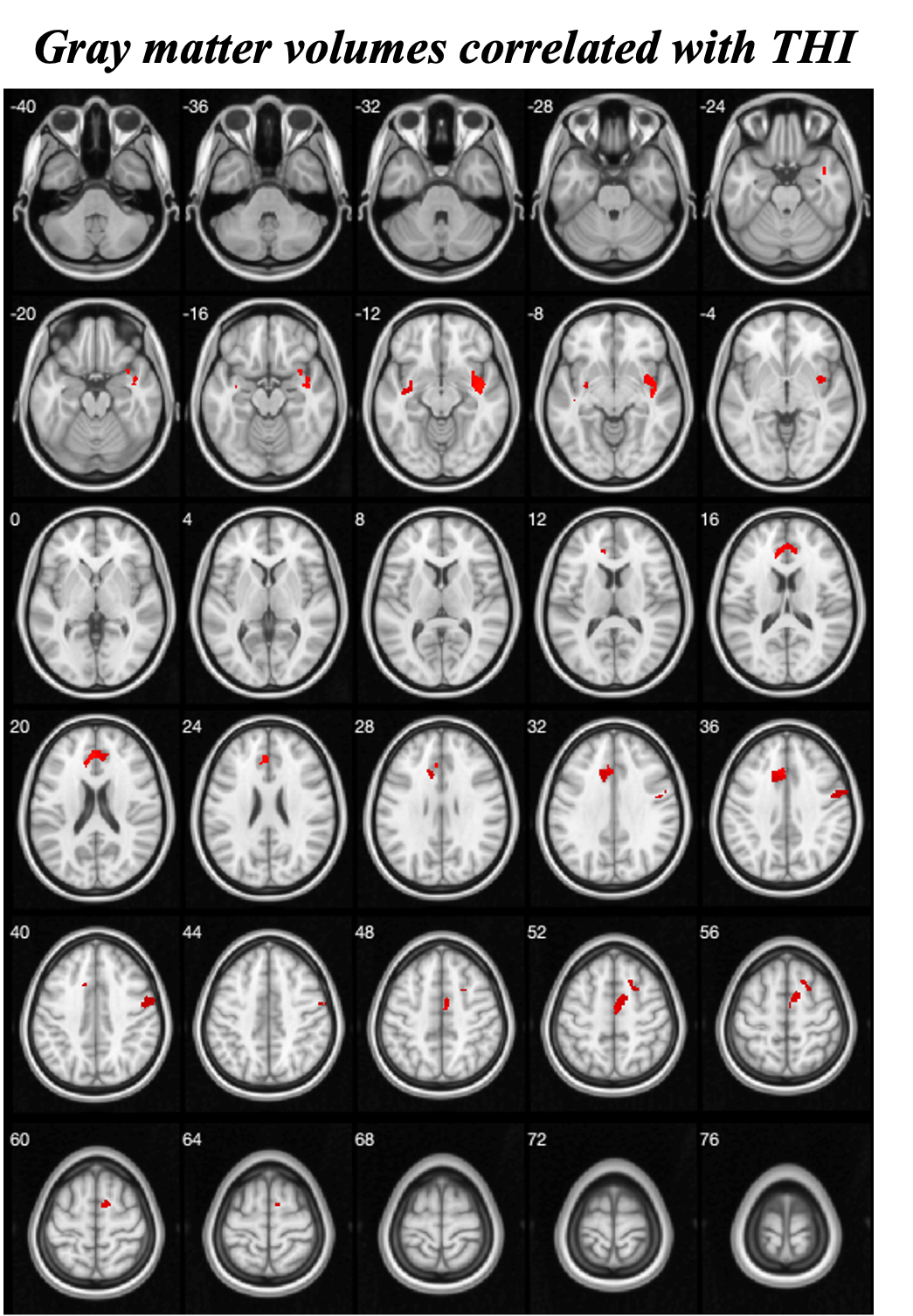

Supplement: Supplementary file 2 [file Image_2.TIFF]
